# Development and validation of a multiple-choice test for head and neck ultrasound certification

**DOI:** 10.1007/s00405-025-09533-3

**Published:** 2025-07-12

**Authors:** August Krogh Mikkelsen, Julian Kuenzel, Lisa A. Orloff, Merry E. Sebelik, Andrew McQueen, Gitta Madani, Lars Konge, Tsung-Lin Yang, Jacob Melchiors, Johannes Weimer, Tobias Todsen

**Affiliations:** 1 Department of Otorhinolaryngology, Head and Neck Surgery and Audiology, Copenhagen University Hospital - Rigshospitalet, Inge Lehmanns vej 8, Copenhagen, 2100 Denmark; 2 Department of Clinical Medicine, University of Copenhagen, Copenhagen, Denmark; 3 Department of Otorhinolaryngology, Head and Neck Surgery, University Hospital Regensburg, Regensburg, Germany; 4 Department of Otolaryngology-Head & Neck Surgery, Stanford University School of Medicine, Stanford, CA USA; 5 Department of Otolaryngology-Head and Neck Surgery, Emory University School of Medicine, The Winship Cancer Institute at Emory University, Atlanta, GA USA; 6 Department of Radiology, Freeman Hospital Newcastle upon Tyne Hospitals NHS Foundation Trust, Newcastle upon Tyne, Newcastle upon Tyne, UK; 7 Department of Radiology, Imperial College Healthcare NHS Trust, London, UK; 8 CAMES– Copenhagen Academy for Medical Education and Simulation, Capital Region of Denmark, Copenhagen, Denmark; 9 Department of Otolaryngology, National Taiwan University Hospital, and National Taiwan University College of Medicine, Taipei, Taiwan; 10 Rudolf Frey Learning Clinic, University Medical Center of the Johannes Gutenberg University Mainz, Mainz, Germany; 11 Department of Medicine I, University Medical Center of the Johannes Gutenberg University Mainz, Mainz, Germany

## Abstract

**Purpose:**

This prospective trial aimed to develop and gather validity evidence for a theoretical test in head and neck ultrasound (HNUS).

**Methods:**

Seven HNUS experts from Europe, North America, and Asia participated in a Delphi study to reach consensus on multiple choice test (MCT) items. Novices (*n*=56) and experienced HNUS operators (*n*=22) were then invited to take the full MCT. Based on their answers, an item-response analysis selected the MCT items with the best performance. Generalizability theory determined the number of MCT items sufficient for certification. An ANOVA test examined the MCT’s ability to distinguish novices from experts and contrasting groups’ standard setting was used to establish a cut-off test score. A group of physicians (*n*=23) was tested at a formal ultrasound course, and pass-fail consequences for the final test were evaluated.

**Results:**

Over three Delphi rounds, 64 items were revised and 21 were excluded, yielding 106 items. The item-response analysis found nine items with low discrimination that were excluded based on the MCQ answers from 78 novices and experienced physicians who had taken the test. The final 97 test items had a high internal consistency reliability of 0.97, and an MCT with 15 items was found sufficient for certification purposes. The MCT could significantly discriminate between novices (mean 51.1, SD 13.8) and experienced participants (mean 92.0, SD 3.1), *p* < 0.001. A pass-fail score of 83 was established. At a formal introduction ultrasound course, 57% of the participants passed the MCT at the established pass-fail score.

**Conclusion:**

The developed MCT for HNUS, based on international expert consensus, has multiple sources of validity evidence to support its use as part of a thorough certification process.

**Supplementary Information:**

The online version contains supplementary material available at 10.1007/s00405-025-09533-3.

## Introduction

The use of head and neck ultrasound (HNUS) by clinicians has increased over the past decade. While patients are still referred to the radiology department for ultrasound examinations, an increasing number of otolaryngologist-head and neck surgeons are using ultrasound in the outpatient clinic or in a surgical setting [1–3]. Traditional approaches to certification of medical practitioners in diagnostic ultrasound have relied on time-based educational methods with a fixed number of ultrasound examinations required [4–6]. However, this approach does not consider the different learning curves among physicians, where competency-based training that relies on skills assessment is instead recommended [7–9].

Competency evaluation should include both theoretical knowledge testing and direct assessment of performance in a clinical setting [10–13]. Research has developed, evaluated, and validated the Objective Structured Assessment of Ultrasound Skills (OSAUS) [14, 15] and Direct Observation of Procedural Skills (DOPS) [16] as assessment tools to evaluate HNUS performance. However, theoretical knowledge assessment tools with sufficient validity evidence are still lacking for certification in HNUS. Multiple-choice tests (MCT) are recommended to standardize theoretical head and neck ultrasound knowledge assessment as they are reproducible and cost-effective [4, 17]. Multiple institutions emphasize the importance of adequate theoretical knowledge for ultrasound practitioners [4, 5, 18, 19]. This study, therefore, aimed to develop and collect validity evidence for an MCT covering the basic theoretical knowledge needed for HNUS.

## Methods and materials

We aimed to develop an MCT covering essential theoretical HNUS knowledge. Messick’s theoretical framework was used to explore the five sources of validity evidence: content, response process, internal structure, relationship to other variables, and consequences [20].

**Fig. 1:**
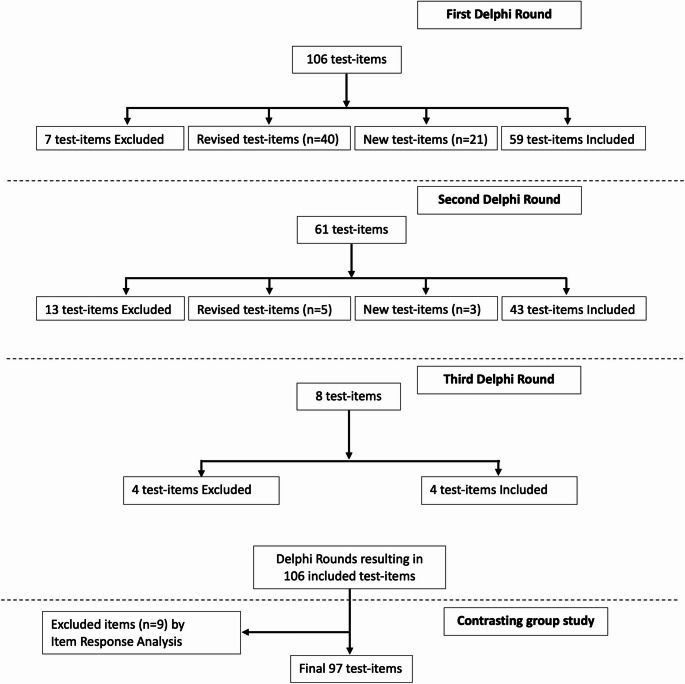
Flowchart of the Delphi-like process. Flowchart of the Delphi-like process with excluded, revised, new, and included test items. 97 items were included after item response analysis based on data from the experimental study with novices and experienced HNUS operators

The study consisted of three phases (Fig. 1) to ensure the collection of all five sources of validity evidence: (1) a Delphi study to obtain expert consensus on the content of test items, (2) an experimental study to select the number of test items needed for a reliable test, and ensure the discriminative ability of the test, and (3) assessment of the consequences of implementing the test at a formal HNUS ultrasound course.

### Expert consensus of test items through a modified Delphi study

Essential theoretical HNUS knowledge was identified before the first Delphi round by reviewing existing scientific literature, relevant textbooks, content from ultrasound courses, and recommendations provided by multiple international institutions [4, 5, 18, 19, 21–25]. The first author developed the test items in collaboration with a committee consisting of an otolaryngologist-head and neck surgeon with expertise in ultrasound, a physician experienced in pre-and postgraduate ultrasound training, and a professor in Medical Education. All test items had one single best answer and two distractors [26]. The Delphi-like process used was a web-based, semi-anonymous, structured methodology designed to collect information from a panel of experts. This approach ensured equal opportunity for all experts to contribute independently, preventing individual dominance and minimizing bias in the consensus process [27]. All data was collected via online survey distribution tools and was only accessible to the steering committee. Seven HNUS experts (five otolaryngologist-head and neck surgeons and two radiologists) representing the United States (*n* = 2), United Kingdom (*n* = 2), Taiwan (*n* = 1), Germany (*n* = 1), and Denmark (*n* = 1) participated in the multi-round Delphi survey. All the experts met, at a minimum, the following predefined criteria: consultants in radiology or otolaryngology–head and neck surgery and performing HNUS examinations regularly, active researchers in ultrasound, and involved in HNUS postgraduate training [28].

The steering committee pre-defined the test item inclusion criteria, requiring 6 out of 7 experts (80%) to evaluate the specific test item as essential by giving it a scale of 4 or 5. If a test item obtained lower scores and revisions were suggested by the expert panel or the steering committee, the steering committee would incorporate the proposed changes and present the revised question for re-evaluation in the subsequent Delphi round. The steering committee did not influence the content of the test once the Delphi-like process had begun but collected, reviewed, analyzed, and implemented the expert panel’s comments, revisions, and Likert-scale ratings for each Delphi round. The steering committee also developed new test items based on the expert panel’s suggestions. Test items that did not meet the inclusion criteria were discarded. While the experts were aware of the expert panel’s composition, individual rating responses remained anonymous and were accessible only to the steering committee.

In the first round of the Delphi-like process, the experts were provided with all the test items developed by the steering committee. The experts were then asked to comment on the test items’ phrasing, clarity, and relevance for HNUS. The expert panel was asked to rate each item on a Likert scale from 1 to 5 (1 = Not relevant, 3 = Relevant - but not essential, 5 = Essential) to ensure the relevancy of the test items. If the experts did not find a test item(s) sufficient for an MCT as part of a certification process, they were required to comment on what they thought needed to be added for the test item(s) to be sufficient. The experts were encouraged to propose new test items, provide relevant ultrasound images or videos, or suggest topics to be included in the theoretical test to ensure it would cover essential HNUS knowledge.

In the second and third rounds, rephrased and new test items were (re-)evaluated by the expert panel. The expert panel was asked to comment and critique the test items by following the framework from round one, as stated above. Finally, the experts stated whether they thought an item was essential and, therefore, should be in the final test. The test item inclusion criteria were the same as in the first round.

### Gathering further validity evidence for the test

The study’s second phase commenced following the Delphi rounds’ completion, and expert consensus on the test content was achieved (i.e., validity evidence for *content*). Here, the test was administered to a group of novices (*n*=56) and a group of physicians with HNUS competence (*n*=22). For the novice group, we invited medical students from Danish and German universities without prior HNUS experience through social media to participate in the study. The HNUS experienced group had formal HNUS training and was acknowledged by the expert panel as someone who would expect to pass the test. Both groups of participants took the test online without any time limit.

For the third phase of the study, we aimed to explore the test consequences. The revised test was therefore taken by 23 participants during a formal HNUS course where they had 45 min to complete the test without access to help. The participants were practicing physicians in radiology or otolaryngology–head and neck surgery and did not use HNUS consistently.

### Statistics

All data were collected via online survey distribution tools (SurveyMonkey and REDcap (v. 13.7.14)) and were only accessible to the research group. Results were transferred to MS Excel for the steering committee to analyze. Statistical analyses were performed using SPSS (v. 29.0.1.0) and G String Software. All statistics were considered significant at a 5% significance level.

A Wilcoxon rank sum test was performed to test significant differences in Likert scale ratings based on the specialty of the expert panel. The item quality was evaluated by performing an item response analysis to assess the internal structure of the test. Here, the item difficulty index is the proportion of test takers who answer an item correctly, and the item discriminatory index is the individual item’s predictive ability in relation to the total test score. Test items were classified by their item difficulty and point biserial discrimination from levels one to four. Level I and II test items made the final test. Level III and IV test items were discarded [13].

The internal consistency reliability of the test was calculated using the Cronbach alpha reliability coefficient [29, 30]. Additionally, generalizability theory and decision studies were employed to estimate reliability across different numbers of test items for a final test to assess theoretical HNUS knowledge [31]. The test scores discriminatory abilities between expert- and novice groups were explored with an analysis of variance (ANOVA) to gather “relations to other variables” validity evidence.

**Fig. 2:**
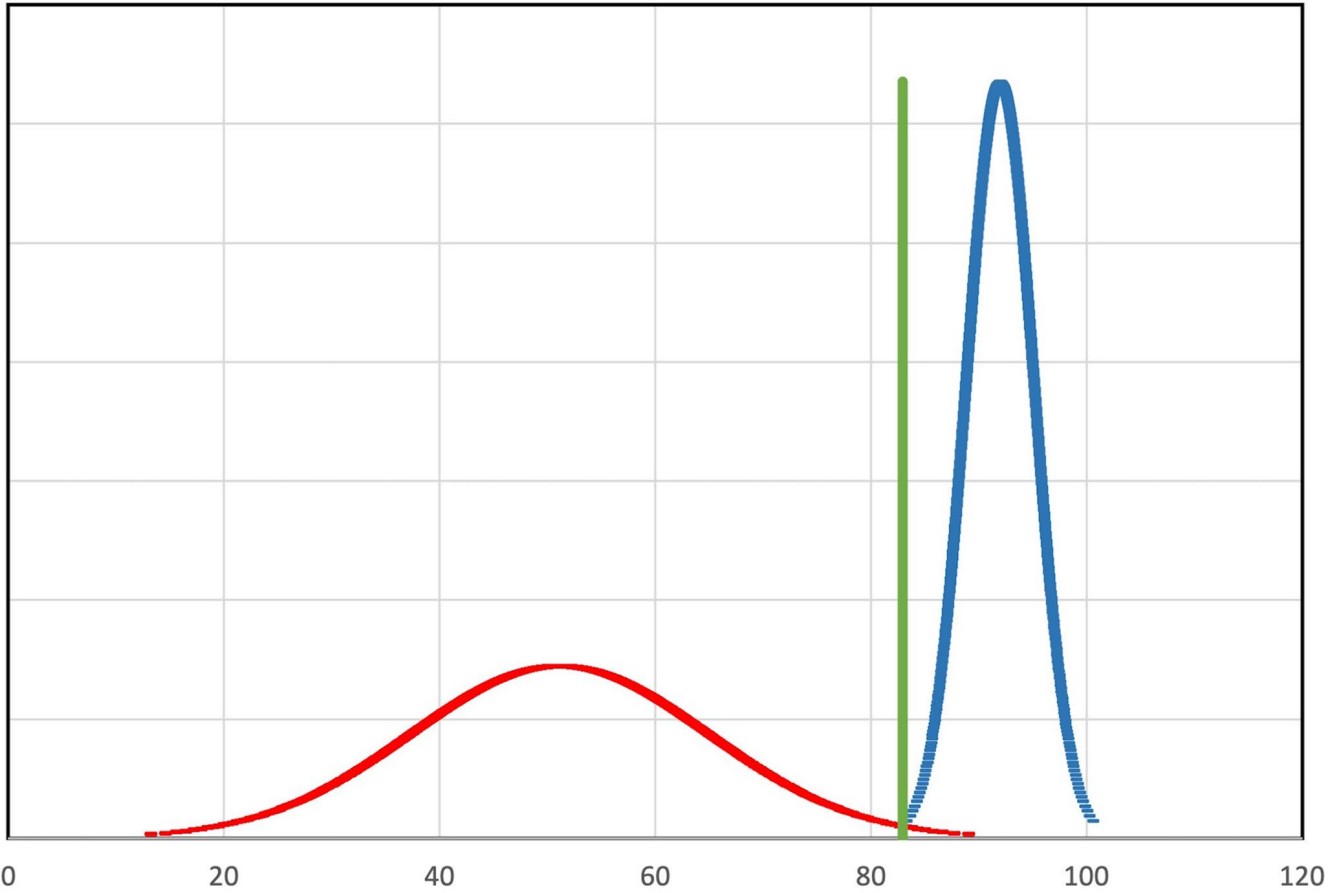
Multiple-choice test score distribution of novices and experienced. Multiple-choice test (MCT) score(x-axis) distribution of novices(red) and experienced(blue). The pass-fail score was established by the contrasting groups’ method. The intersect(green) between the score curves of the novices and the experienced visualizes the pass-fail score of 83

The contrasting groups’ standard setting was used to establish a pass-fail score and analyze the consequences of the test. The two groups and their respective mean scores were visualized (Fig. 2), where the intercept between the distributions of the two groups represents the pass-fail cut-off score [32].

## Results

Seven experts participated in the Delphi study and completed three Delphi rounds before they achieved consensus regarding the final MCT items. Initially, 106 MCT items were distributed to the expert panel. After the first Delphi round, 59 test items were included, 7 test items were excluded, 40 test items were revised, and the experts suggested 21 new test items. 61 items were distributed to the expert panel in the second round. The second round had 43 test items included, 3 new test items, 5 revised test items, and 13 test items excluded. In the third round, 8 Items were distributed to the expert panel − 4 test items were excluded, and 4 test items were included. The three Delphi rounds resulted in an assessment tool consisting of an MCT item bank with 106 test items testing essential HNUS knowledge (see Fig. 1). A Wilcoxon rank sum test showed no statistical differences in scores between specialties in the Delphi rounds (*p* = 0.65).

**Fig. 3:**
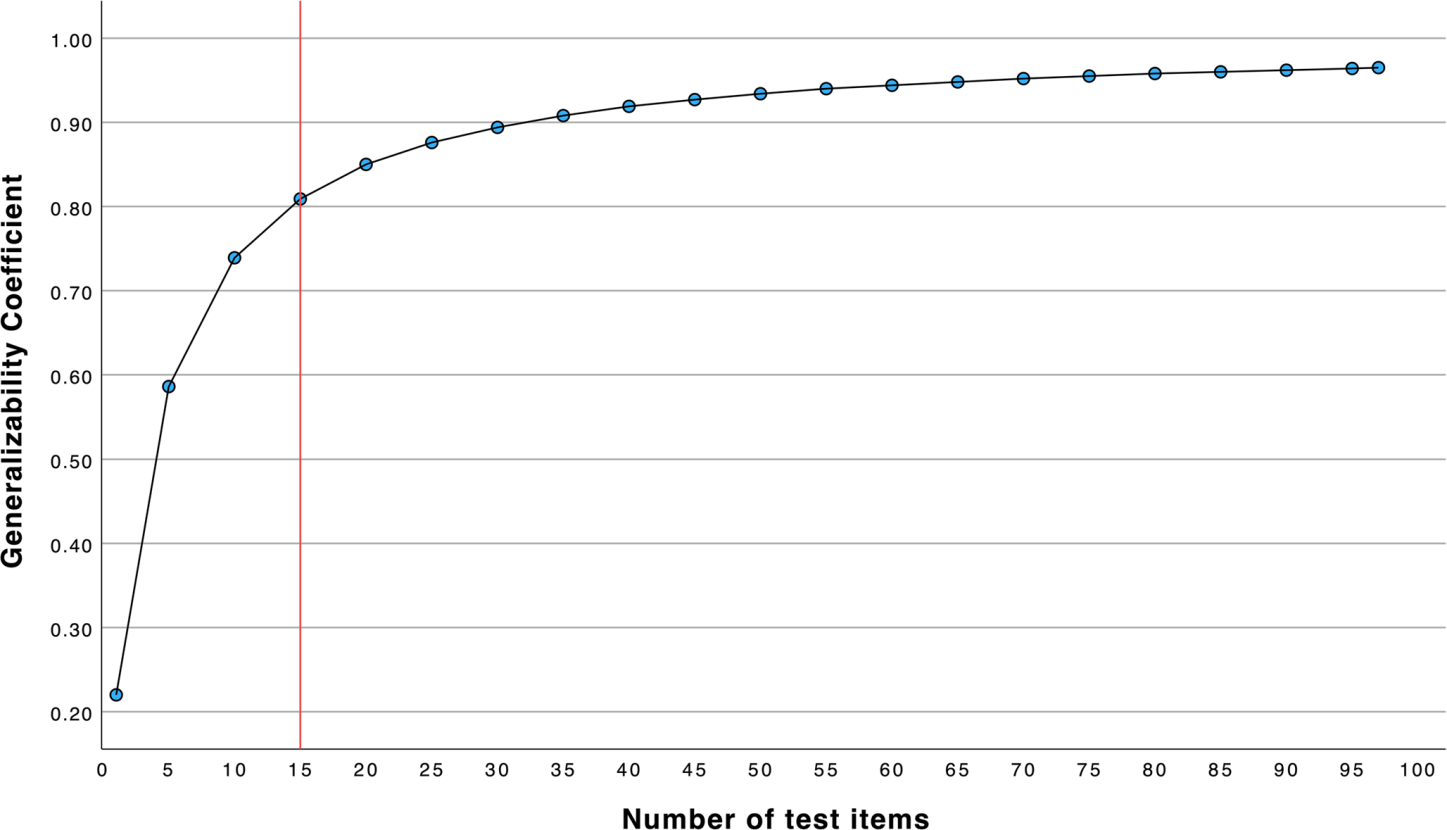
Generalizability coefficient for different number of test items. Generalizability coefficient for different number of test items, achieved through a Decision study. The red line indicates that sampling 15 test items achieves a Generalizability coefficient above 0.8 which suffices for certification purposes

The test items were classified into four groups based on their item difficulty and the item discrimination (point biserial) [13] (Table 1). Level III and IV test items (*N* = 9) were discarded, as these are the least effective test items, meaning the final MCT item bank consists of 97 level I and II test items. The internal consistency reliability for the final 97 test items was very high (Cronbach’s Alpha = 0.97) (Fig. 3). A decision study showed that a sample of 15 test items was needed to obtain a G-Coefficient of 0.8, meaning 15 of the final test items are sufficient for certification purposes.

**Table 1:** Classification of items based on item difficulty and item discrimination (point biserial)

Classification	Item difficulty	Item discrimination (point biserial)	Number of items
Level I (Best items)	0.45–0.75	> 0.20	*n* = 67
Level II (Easier items)	0.76–0.91	> 0.15	*n* = 30
Level III (Difficult items)	0.25–0.44	> 0.10	*n* = 3
Level IV (extremely difficult or easy)	< 0.24 or > 0.91	Any discrimination	*n* = 6

**Fig. 4:**
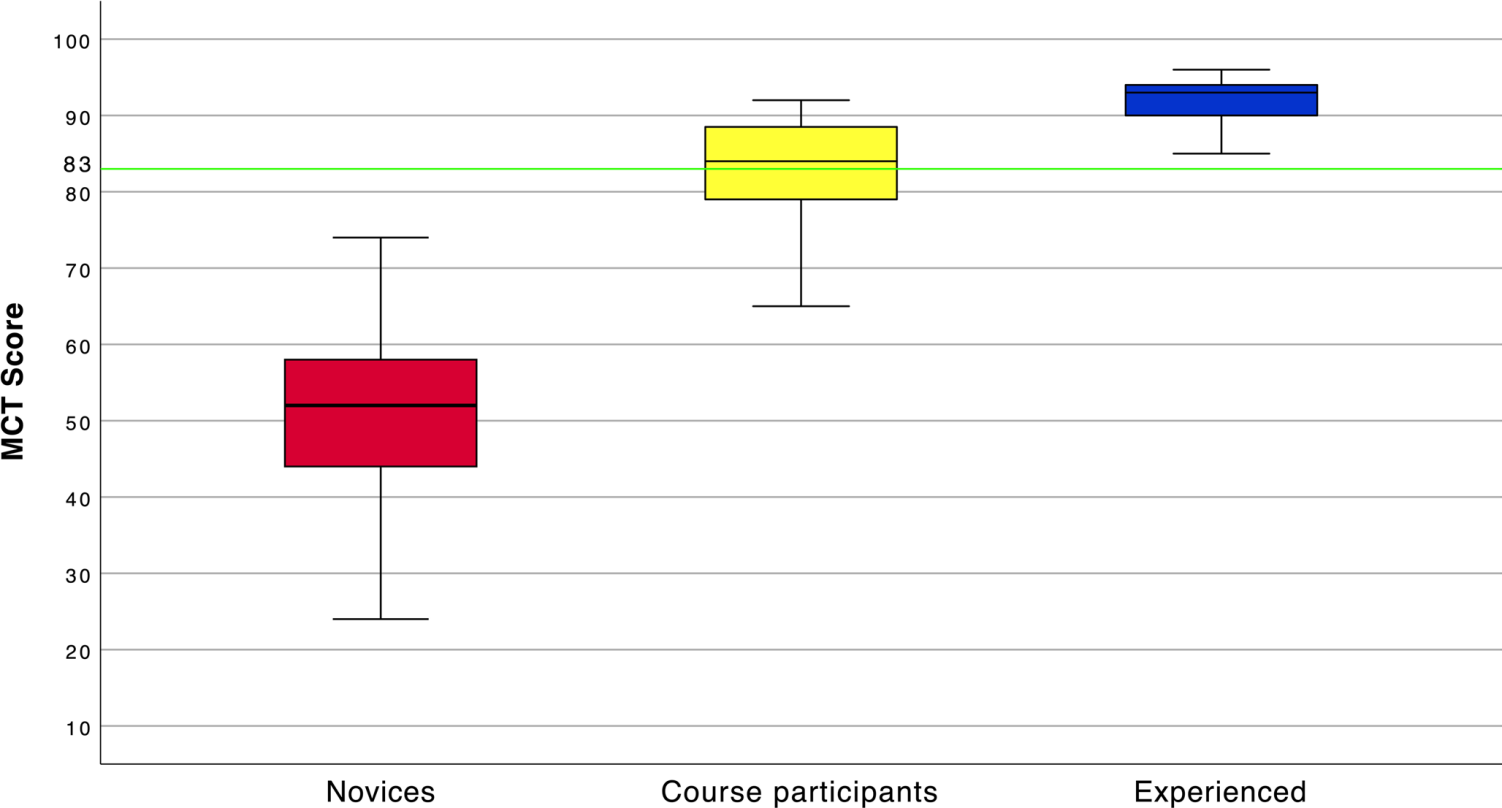
Differences in multiple-choice test scores between groups of head and neck ultrasound operators. Boxplot visualizing the differences in multiple-choice test scores between groups of novice head and neck ultrasound (HNUS) operators, physician course participants at a formal HNUS course, and experienced HNUS operators. Highlighted in green is the Pass-fail score of 83. MCT: Multiple-choice test

Boxplots in Fig. 4 visualize the differences in test scores between the novices, course participants, and experienced group, and Table 2 compares the mean scores between the three groups. The groups performed significantly differently (*p* < 0.001).

**Table 2:** Comparison of MCT mean scores between experienced, novices, and course participants

Comparison between groups (Mean MCT score ± SD)	Absolute difference in mean score
Experienced (92.0 ± 3.1)– Novices (51.1 ± 13.8)	40.9
Experienced (92.0 ± 3.1)– Course Participants (81.3 + 11.4)	10.7
Course Participants (81.3 + 11.4)– Novices (51.1 ± 13.8)	30.2

The contrasting groups’ method established a pass-fail score of 83, meaning participants had to answer 85% of the MCT items correctly to pass the test (Fig. 2). At this pass-fail-score, 1 novice passed the test, while 0 experienced failed the test. Of the 23 international ultrasound course participants, 13 (57%) passed the test.

## Discussion

This study obtained international expert consensus on an MCT to assess HNUS competence. The MCT could significantly discriminate between competence levels and demonstrated a high reliability in test scores. A cut-off score was established and demonstrated to be useful for participants in a formal ultrasound course to ensure competency-based certification.

This is the first study to develop and validate a MCT that can be used for theoretical assessment of HNUS competency. We conducted a comprehensive study, including a Delphi study combined with two experimental trials to select the best discriminative test items and explore the practical consequences of implementing the MCT. In our Delphi-like process, the recruited multiple international experts ensured that the content of our assessment tool is relevant for assessment of HNUS competence globally. Our semi-anonymous online study ensured that the experts could express their ideas without interference.

Another strength of this study is the combination of a Delphi study with two experimental trials to explore all sources of validity evidence according to the Messick validity framework (Table 3).

**Table 3:** Sources of validity evidence based on Messick’s framework of validity

Source of validity evidence	Definition	Gathered validity evidence
1. Content	The content of the test should measure what is intended. In this study theoretical competence in HNUS	Development based on relevant literature, publications, expert input and through expert consensus in the Delphi-like process
2. Response process	Integrity and quality of the test is observed and preserved at all times.	The test setting was equal to all using a online link format.
3. Internal structure	Referring to the internal consistency reliability of the test items, where similar scores should be achieved when measuring the same construct.	Internal consistency reliability: - Cronbach’s alpha = 0.97 Item quality was explored by performing an item response analysis
4. Relationship to other variables	Test scores should correlate to known variables, here level of experience	We found significant test score difference between the novice group(*n* = 56) had a mean ± SD test score of 51.1 ± 13.8, while the experienced group(*n* = 22) had a mean ± SD test score of 92.0 ± 3.1. Participants at the international HNU course had a mean ± SD of 81.3 ± 11.4.
5. Consequences	Refers to the standard setting of a pass/fail score to support interpretation of test results.	Contrasting groups’ standard setting was used to set a pass/fail score of 83. 57% of the participants at the international HNU course passed the test.

We believe the results are generalizable to other settings, as more than 100 volunteer participants from multiple countries were included. The final item bank also ended with 97 test items, much more than the minimum recommendations of 40 test items for a three-option MCT [13]. Delphi studies have been used in several other medical specialties to develop and gather validity evidence for MCT item banks [33–35]. Other studies have utilized informal/unstructured conversational interviews for the item development process [34]. Our study method ensures process transparency and the opportunity for all content experts to provide feedback on established themes and contribute new test items during the Delphi rounds. The latter approach is a strength in our study, as it decreases the probability that specific content experts were superseded by others, minimizing bias in the consensus process. At the same time, it also ensures the coverage and elaboration of essential content for a HNUS MCT.

Our study has some limitations. For the standard setting, the novice group comprised medical students with no experience in HNUS. This could serve as a limitation as the reliability score might have been increased artificially and lowered the final cut-off score. However, when tested on delegates at a formal ultrasound course, the MCT still demonstrated good discriminative ability, thereby minimizing the possibility that this would have implications in a real-world setting.

The expert selection might serve as another potential limitation of our study, as a larger and more balanced expert panel, comprising an equal number of otolaryngologist-head and neck surgeons and radiologists, might have influenced the outcome of the MCT item bank differently. However, our analysis showed that there were no statistical differences in scores between specialties in the Delphi rounds.

As clinician-conducted ultrasound has become widespread among various specialties such as otolaryngologists-head and neck surgeons, endocrinologists, and pediatricians, it is essential to ensure that operators demonstrate adequate competency before conducting unsupervised scans [4].

Based on the results from this study, a validated MCT has been developed to assess necessary theoretical knowledge in HNUS. The MCT provides a standardized and reliable method for evaluating theoretical knowledge. At an international formal HNUS course, 13 of 23 (57%) passed the test. This further emphasizes that the test is reliable and applicable in a real-life setting.

The MCT can be integrated into formal HNUS courses, ideally serving as a prerequisite before further objective assessment with a validated tool e.g. the OSAUS [14] or DOPS [16] to ensure a comprehensive and thorough evaluation of basic HNUS competence for certification. This approach would ensure a standardized international assessment of both theoretical and practical ultrasound competency in HNUS.

As this study focused on developing an MCT to assess the theoretical knowledge of diagnostic ultrasound skills, further research should focus on validating assessment tools for ultrasound-guided biopsies of the head and neck, which is also an essential part of the diagnostic work-up [36]. 

## Conclusion

This study developed an MCT item bank to assess HNUS competence based on international expert consensus. It demonstrated that it could discriminate between competence levels and was highly reliable in test scores. A pass-fail cut-off score was established and found to be feasible for implementation during or before formal head and neck ultrasound training courses for certification.

A selection of sample items can be seen in the supplementary files.

## Supplementary Information

Below is the link to the electronic supplementary material.

ESM 1 (PNG 119 KB)

ESM 2 (PNG 319 KB)

## References

[1] Russell MD, Orloff LA (2022) Ultrasonography of the thyroid, parathyroids, and beyond. Hno 70:333–344. 10.1007/s00106-022-01162-0 35364686 10.1007/s00106-022-01162-0 PMC8974803

[2] Warm JJ, Melchiors J, Kristensen TT et al (2024) Head and neck ultrasound training improves the diagnostic performance of otolaryngology residents. Laryngoscope Investig Otolaryngol 9:e1201. 10.1002/lio2.1201 38362178 10.1002/lio2.1201 PMC10866603

[3] American Academy of otolaryngology-head and neck surgery position statement: Surgeon performed neck ultrasound. In https://www.entnet.org/resource/position-statement-surgeon-performed-neck-ultrasound/

[4] Todsen T, Ewertsen C, Jenssen C et al (2022) Head and neck ultrasound - EFSUMB training recommendations for the practice of medical ultrasound in Europe. Ultrasound Int Open 8:E29–e34. 10.1055/a-1922-6778 36212171 10.1055/a-1922-6778 PMC9546639

[5] Radiologists TRCo Ultrasound training recommendations for medical and surgical specialties. https://www.bmus.org/static/uploads/resources/bfcr173_ultrasound_training_med_surg.pdf

[6] Todsen T (2017) Surgeon-performed ultrasonography. Dan Med J 64 PMID: 29115210. https://www.ugeskriftet.dk/dmj/surgeon-performed-ultrasonography 29115210

[7] Jang TB, Ruggeri W, Dyne P et al (2010) The learning curve of resident physicians using emergency ultrasonography for cholelithiasis and cholecystitis. Acad Emerg Med 17:1247–1252. 10.1111/j.1553-2712.2010.00909.x 21175524 10.1111/j.1553-2712.2010.00909.x

[8] Frank JR, Mungroo R, Ahmad Y et al (2010) Toward a definition of competency-based education in medicine: a systematic review of published definitions. Med Teach 32:631–637. 10.3109/0142159X.2010.500898 20662573 10.3109/0142159X.2010.500898

[9] Miller GE (1990) The assessment of clinical skills/competence/performance. Acad Med 65:S63–67. 10.1097/00001888-199009000-00045 2400509 10.1097/00001888-199009000-00045

[10] Kumar A, Kugler J, Jensen T (2019) Evaluation of trainee competency with Point-of-Care ultrasonography (POCUS): a conceptual framework and review of existing assessments. J Gen Intern Med 34:1025–1031. 10.1007/s11606-019-04945-4 30924088 10.1007/s11606-019-04945-4 PMC6544692

[11] Hawkins R, Swanson D (2008) Using written examinations to assess medical knowledge and its application. Pages 42–59

[12] Höhne E, Recker F, Dietrich CF et al (2022) Assessment methods in medical ultrasound education. Front Med 9. 10.3389/fmed.2022.871957 10.3389/fmed.2022.871957 PMC9218354 35755059

[13] Downing S, Yudkowsky R (2009) Assessment in health professions education. New York and London: Routledge and Taylor & Francis

[14] Tolsgaard MG, Todsen T, Sorensen JL et al (2013) International multispecialty consensus on how to evaluate ultrasound competence: a Delphi consensus survey. PLoS ONE 8:e57687. 10.1371/journal.pone.0057687 23469051 10.1371/journal.pone.0057687 PMC3585207

[15] Todsen T, Melchiors J, Charabi B et al (2018) Competency-based assessment in surgeon-performed head and neck ultrasonography: A validity study. Laryngoscope 128:1346–1352. 10.1002/lary.26841 28868625 10.1002/lary.26841

[16] Weimer JM, Rink M, Müller L et al (2023) Development and integration of DOPS as formative tests in head and neck ultrasound education: proof of concept study for exploration of perceptions. Diagnostics 13:661 36832149 10.3390/diagnostics13040661 PMC9954978

[17] Schuwirth LW, van der Vleuten CP (2003) ABC of learning and teaching in medicine: written assessment. BMJ 326:643–645. 10.1136/bmj.326.7390.643 12649242 10.1136/bmj.326.7390.643 PMC1125542

[18] American Institute of Ultrasound in Medicine A (2023) Training Guidelines for Physicians Who Perform and/or Interpret Diagnostic Ultrasound Examinations. In: Mar 10, 2023 edn. AIUM

[19] (2014) AIUM practice guideline for the performance of ultrasound examinations of the head and neck. J Ultrasound Med 33:366–382. 10.7863/ultra.33.2.366 10.7863/ultra.33.2.366 24449746

[20] Messick S (1995) Validity of psychological assessment: validation of inferences from persons’ responses and performances as scientific inquiry into score meaning. Am Psychol 50:741–749. 10.1037/0003-066X.50.9.741

[21] Case S, Swanson D (2002) Constructing written test questions for the basic and clinical sciences. Journal: National Board of Examiners https://www.researchgate.net/publication/242759434_Constructing_Written_Test_Questions_For_the_Basic_and_Clinical_Sciences

[22] Thomas M, Haladyna MCR (2013) Developing and Validating Test Items. Routledge. 10.4324/9780203850381

[23] Orloff LA (2016) Book: Head and neck ultrasonography: essential and extended applications. Plural Publishing

[24] Ahuja ATER (2000) Practical head and neck ultrasound. Cambridge University Press

[25] AIUM (2014) AIUM practice guideline for the performance of ultrasound examinations of the head and neck. J Ultrasound Med 33:366–382. 10.7863/ultra.33.2.366 24449746 10.7863/ultra.33.2.366

[26] Raymond MR, Stevens C, Bucak SD (2019) The optimal number of options for multiple-choice questions on high-stakes tests: application of a revised index for detecting nonfunctional distractors. Adv Health Sci Educ 24:141–150. 10.1007/s10459-018-9855-9 10.1007/s10459-018-9855-9 30362027

[27] Hasson F, Keeney S, McKenna H (2000) Research guidelines for the Delphi survey technique. J Adv Nurs 32:1008–1015 11095242

[28] Palter VN, MacRae HM, Grantcharov TP (2011) Development of an objective evaluation tool to assess technical skill in laparoscopic colorectal surgery: a Delphi methodology. Am J Surg 201:251–259. 10.1016/j.amjsurg.2010.01.031 20832048 10.1016/j.amjsurg.2010.01.031

[29] Downing SM (2004) Reliability: on the reproducibility of assessment data. Med Educ 38:1006–1012. 10.1111/j.1365-2929.2004.01932.x 15327684 10.1111/j.1365-2929.2004.01932.x

[30] Cronbach LJ (1951) Coefficient alpha and the internal structure of tests. Psychometrika 16:297–334. 10.1007/BF02310555

[31] Bloch R, Norman G (2012) Generalizability theory for the perplexed: a practical introduction and guide: AMEE guide 68. Med Teach 34:960–992. 10.3109/0142159X.2012.703791 23140303 10.3109/0142159X.2012.703791

[32] Jørgensen M, Konge L, Subhi Y (2018) Contrasting groups’ standard setting for consequences analysis in validity studies: reporting considerations. Adv Simul 3:5. 10.1186/s41077-018-0064-7 10.1186/s41077-018-0064-7 PMC5845294 29556423

[33] Jørgensen M, Savran MM, Christakopoulos C et al (2019) Development and validation of a multiple-choice questionnaire-based theoretical test in direct ophthalmoscopy. Acta Ophthalmol 97:700–706. 10.1111/aos.14065 30816642 10.1111/aos.14065

[34] Savran MM, Clementsen PF, Annema JT et al (2014) Development and validation of a theoretical test in endosonography for pulmonary diseases. Respiration 88:67–73. 10.1159/000362884 24853171 10.1159/000362884

[35] Savran MM, Hansen HJ, Petersen RH et al (2015) Development and validation of a theoretical test of proficiency for video-assisted thoracoscopic surgery (VATS) lobectomy. Surg Endosc 29:2598–2604. 10.1007/s00464-014-3975-y 25427417 10.1007/s00464-014-3975-y

[36] Todsen T, Bennedbaek FN, Kiss K et al (2021) Ultrasound-guided fine-needle aspiration biopsy of thyroid nodules. Head Neck 43:1009–1013. 10.1002/hed.26598 33368812 10.1002/hed.26598

