# Supplementary figures and images for: Development and validation of a multiple-choice test for head and neck ultrasound certification
Source: Eur Arch Otorhinolaryngol. 2025 Jul 12;282(9):4825–33. doi: 10.1007/s00405-025-09533-3 (PMC12423143; doi:10.1007/s00405-025-09533-3)

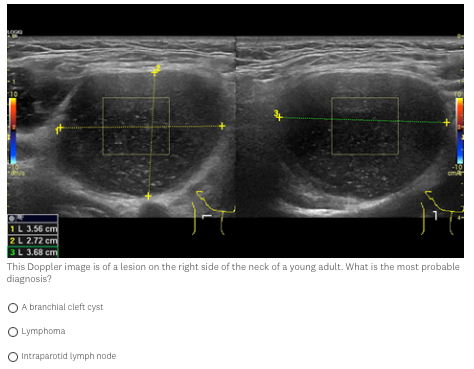

Supplement: Supplementary file 1 — (PNG 119 KB) [file 405_2025_9533_MOESM1_ESM.png]

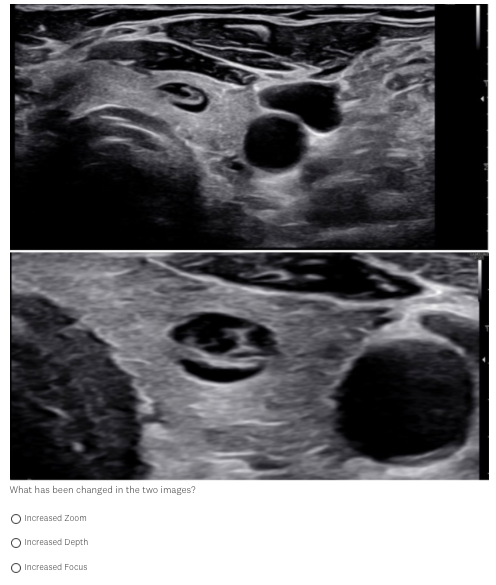

Supplement: Supplementary file 2 — (PNG 319 KB) [file 405_2025_9533_MOESM2_ESM.png]
